# Editors’ Table

**Published:** 1857-05

**Authors:** 


### Editors' Table.

ETHNOLOGY OF CENTRAL AFRICA.—Facts new to most of our readers, illustrative of the physical and mental characteristics of the people of Central Africa, are presented in a small and quite an attractive volume, by the Rev. T. I. Bowen, entitled, "Adventures and Missionary Labors in Several Countries in the Interior of Africa, from 1849 to 1856." The work is issued from the Southern Baptist Publication Society, Charleston, S. C. An infusion of the blood of the Caucasian stock in that of the negro, prevails to a much greater extent, and dates from a remoter period than geographers and ethnologists have hitherto taught us. Mr. Bowen divides the people of Central Africa into three classes, viz., typical negroes, mulattoes, and black men with a European cast of countenance. Eastern and Southern Africa afford other varieties, of which we shall not speak at present.

1. The typical negro, as every one knows, is distinguished by his inferior organization. He has great endurance of fatigue, and of exposure to heat and moisture, and suffers less pain from blows, wounds, or surgical operations, than the white man. His intellect and especially his reasoning faculties are weak, his moral perceptions low, and his animal feelings strong. "He appears to be a stranger to modesty, doing and allowing things with brutal apathy, which other races cannot tolerate. I doubt whether any negro of this class has ever felt disgust, or ever will. They are naturally incapable of refined feelings."

2. Many of the people of the interior tribes, including the Pulohs, and a few of the Yarubas, Mandingoes, and Kroomen, are mulattoes, or the progeny of a union of the white man and black races or typical negro, in various degrees and shades. In proof of this, Mr. Bowen adduces the following facts: 1. Their color varies from dark to very bright. Some of the Pulohs or Fellalahs cannot have more than one-eighth of negro blood, if we can judge from their color. 2. Their hair, though woolly, is long and bushy, like that of other mulattoes. Mr. B. has seen one woman nearly black, with soft silky hair. Some have a sandy tint of beard and hair, as if their ancestors were red-headed. The author saw one with bright blue eyes. Lander saw one on the Niger. 3. Their features, noses, lips, skull, &c., are cast more or less in the European mould. Their hands and feet are small and elegantly formed. 4. The language of the Pulohs, of which the author collected about three hundred phrases, containing one thousand words or more, is not African or Shemitic. 5. The Pulohs affirm that their ancestors were white. 6. And finally, we have evidence, mostly of more or less confidence, that the white and negro races have repeatedly come in contact, under circumstances which must have resulted in amalgamation. We shall not follow Mr. Bowen in his speculation on the origin of the negroes in Northern India, nor of their dispersion thence by three emigrations, the one into Eastern Asia, another into Southern India, and the Indian Archipelago, and the third and chief to the southwest, and over into Africa. Adopting this view, it is easy to believe "negroes were intermixed with whites long before they entered Africa, and that mulatto races may have existed in Sudan ever since it was peopled." To this it may be objected, that it is not conformable with ethnological experience to find the type of a race at the very border of the great circle or wide expanse of countries from which it originates, and no remains of it at the disputed original seat. We meet with no approach to the typical negro in Upper India. It is not so with the white or Caucasian stock, some of the finest specimens of which are seen in the mountainous regions, whence it is supposed to have sprung. We may find, it seems to us, an easier solution of the problem in the emigration of people of the white race from Northern Africa, and in the conquest, as is alleged by some of their native historians, of Sudan by the Saracens, in the tenth century. For ages past, Moor and Arab are met with in all parts of Africa, as traders, and as followers of Mahomet, teaching his dogmas, and winning over proselytes.

Two remarks worthy of repetition here, are made by the author. First, that the burning sun and dry air of the desert have not changed the color or the features of the whites who have been there three or four thousand years. Their children are still as white as any in the world. Secondly, the mulatto Pulohs must have been mulattoes centuries ago, and they have intermarried among themselves, "hybrids with hybrids," all the time; otherwise many of them would not still remain as bright-colored as Quadroons, or even brighter. But the Pulohs are physically and mentally a fine race. They show no symptoms of dying out.

The third of the classes above mentioned are black people with European features. This will not surprise those who have lived or travelled in our Southern States of the Union. They must have seen this style of features and a share of corresponding intelligence in the real negro, whose pedigree can be distinctly traced to his slave progenitor from Africa. At Ilorin, Mr. Bowen saw a few robust, handsome, heavily-bearded men, who are called "Bature Dudau," black white men. They differ from the Grecian-faced men, of whom he had spoken a few lines previously, in their being in every way more manly in appearance, and they bear the reputation of being more learned than any other men in the country. One of them, a most noble-looking man, "black but comely," was the chief Alufà, or Doctor of Divinity, in Ilorin. The home of these men appears to have been in Eastern Yoruba from time immemorial, though they are evidently another race. Mr. Bowen supposes that all classes of these black men with European features are the descendants of mulattoes and negroes, who retain the features of the former and the skin of the latter. A vast majority of negroes, both in Guinea and Sudan, belong to this class. In the last-mentioned country he estimates the mulatto population to be from ten to twenty millions. The true or typical negroes are the least numerous of the three, even if we include all the most degraded nations of Guinea. An encouraging prospect is offered in this ethnological view for the future civilization of Central Africa, the most populous and the most fertile division of the whole continent.

"It is not easy, if possible, to ascertain a people's mental powers before they have been developed by exercise, and exhibited in the various pursuits of civilized life. The Yorubas, Pulohs, &c., frequently have a good brain and temperament, in some cases decidedly fine. Very often the reflective faculties are equal to the perceptive, or superior. The Yoruba language is remarkably rich in abstract terms." "Very often there is both an abstract and a concrete noun from the same verbal root. The existence and constant use of terms for the expression of thought are certainly good evidence that the people think. The Yoruba language affords all the terms necessary for a full and clear declaration of the Gospel; as, for instance, a word for God, angel, heaven, hell, sin, guilt, atonement, mediation, repentance, faith, pardon, justification, sanctification, both objective and subjective; a distinct word for each, adoption, salvation, perdition, &c. The reason why they pay so much attention to preaching as constantly reported by the missionaries, is that they understand what the Gospel teaches." The government and laws furnish farther indications of the intellect of these people. But the reverse of the picture exhibits great deficiencies, such as their total want of invention of the commonest mechanical contrivances, even to the making of a crutch, although cripples are common enough. They are almost as deficient in science as the Hottentots, having no weights or uniform measures, and in short, no elementary science of any description. Different from the Yorubas, who display no talent for music, the Pulohs compose and sing fine and noble airs, which would be counted beautiful in any country.

Other interesting details follow respecting the character of the Yoruba people, whose country, we ought to have said before, lies to the west of Sudan, or between this latter and a strip of country whose shores are washed by the Atlantic. Mr. Bowen speaks highly of their natural kindness and gentleness; their polite and courteous demeanor in their intercourse with strangers and with each other; their honesty and truthfulness, and forgiving disposition; their reverential regard for their parents and rulers, and the chastity of their women. But on the other hand we are told, and it is not easy for us to reconcile such contradictions in individual or national character, of their want of conscience, or a sensitive regard for what is right, They lack modesty, and are covetous. although in this particular, the author admits that they have their equals among the whites, and even among too many members and teachers in the churches of civilized countries. Social life in Yoruba is pleasantly described by Mr. Bowen, but we should be travelling from the record were we to follow him any longer. There is a chapter preceding that from which we have borrowed, on the seasons, weather, and diseases of the natives, and of missionaries and other strangers in the Yoruba country, which is germain to our wants, and of which we may be tempted ere long to make use.

---

THE GREAT TOBACCO QUESTION.—IS SMOKING INJURIOUS TO HEALTH?—The London Lancet has opened its columns to a free discussion of the effects produced by the smoking of tobacco on the animal economy. As usual, in all debateable questions, with much pertinent there is a good share of irrelevant matter introduced by the writers in the Lancet, who argue for or against tobacco smoking. The advocates for the use of this weed, say, that no adverse inferences ought to be drawn from the effects of its excessive use or abuse; and they claim for it, at the same time, a soothing and gratefully sedative influence, not incompatible with the preservation of health. On the other side, it is contended that the habitual use of so active and deadly a poison as tobacco is known to be—so deadly, in fact, that there is always danger, and there have been not a few cases of death from its employment in surgery—must necessarily be an abuse. In certain temperaments and constitutions, smoking in any degree is pernicious, and in such cases the use must necessarily be an abuse.

It is not our purpose at this time to argue the question under its several heads—hygienic, therapeutical, pathological, and economic. We shall wait until it has been exhausted in the Lancet, and then make a summary of the leading facts on both sides, unless our London contemporary should perform this office for the benefit of its own readers, and thus anticipate our decision in the matter.

---

THE OPIUM TRADE.—We find it stated in a late periodical, that the revenue derived by the East India Company from opium, in the year 1854–5, was equal to fifteen millions and a half of dollars. This amount is mainly derived from the monopoly enjoyed by the Company in the cultivation and exportation of opium to China, to the people of whom the English present themselves as venders of this deleterious drug, and as in fact poisoners on a grand scale. We have not at hand an estimate of the quantity actually consumed, and the money paid by the Chinese, with such disastrous effects on the health and morals of millions of that people; but the amount, in both instances, must be great when the revenue from it comes up to such a sum as that above mentioned. One of the results of the last war with China, and apparently the chief one contemplated, was to compel the government at Pekin to allow of this wholesale poisoning, which it had previously done all in its power to resist, by prohibiting the importation of opium under heavy penalties. Will the present hostilities at Canton, and destruction of that city, be attended with the effect of still farther favoring the opium trade between China and India? We might ask, also: how many English missionaries and how many Chinese converts to Christianity will make an offset to the demoralization and diseases produced by the English government and English traders by the opium which they have forced into such extensive use in the Chinese empire?

---

SPANISH MEDICINE.—There are now lying before us some numbers of a journal entitled *La Espana Medica*, or "Medical Spain," being the official periodical of the Madrid Chirurgical Academy, edited by Doctor D. Andres del Busto y Lopez. Let us run over the titles of the several departments in one number, that of January 10th, 1857. First, we meet with "*Governmental Medicine*," under which head, Dr. A. del Busto has written a flowery article on the Social Position of the Medical Classes, asking very pertinently why should not physicians, whose preliminary education, intelligence, difficult, extended, and laborious studies and attainments, place them at least on an equality with the members of any other profession or calling, enjoy consideration in the public eye, and be the recipient of honors and rewards, as well as the soldier, the lawyer, or the literati in other departments? Why, in the disposal of public offices, do we not find a physician placed on a line with other individuals? Why should men with the honors of the professorate have less salaries than those who belong to the magistracy? Why should a sanitary director not enjoy equal consideration and regard with a director in military matters, or of other special divisions of public labor?

We give the questions of the Spanish Doctor without attempting ourselves to furnish the answers, which we leave to our readers to fill up for themselves. There is yet another question often asked by our medical brethren in Great Britain and Ireland. Why is it, after a long and laborious struggle with old prejudices and official opposition, in which medical men have taken the largest and most efficient share, and when a sanitary commission of inquiry is appointed, or a sanitary commissioner is made, and a good salary settled on him, that medical men are overlooked in favor of those who are not in the profession, and whose studies, if they ever did study at all, and occupations had been quite foreign from subjects of hygiene and medicine?

Under the next head, "*Medical Miscellany*," there is a continuation of an original article entitled, Brief Considerations on Pain, in a Surgical Point of View, with conclusions, being the results of experiments on this point made by the author, Dr. Ramon Otero, and his colleagues, Valle and Andrey, during their courses in the Faculty of Medicine at Santiago, in the years 1854 and 1855. The use of anæsthetics is discussed at the same time in this paper. Then follows a *Review of Schools*, in which Dr. A. del Busto, after pointing out the fine arrangement of the anatomical amphitheatres of the Faculty of Medicine of Madrid, and the facilities afforded for dissection by a suitable supply of subjects, suggests some improvements: First, a milder temperature in the dissecting rooms, so that students shall not be obliged to drop their scalpels on account of the extreme cold; secondly, a supply of better instruments for dissection, and microscopes always at hand, so as to give the student at any moment an opportunity for examining an organ or tissue, and thus to accustom him to methodical examination by the microscope; and finally, to put on the footing of practical studies, histology, microscopic anatomy, and pathology (meaning most probably morbid anatomy); and that the students of four years' standing shall cultivate more than they do surgical anatomy; engage in some dissections of subjects of the four divisions of the animal kingdom established by Cuvier; and thus be led to unite the study of comparative with that of human anatomy. Could we ask for more than this in young and progressive America?

The third division in the journal is the "*Clinical Section*," in which is a paper on Intermittent Fever, as it occurred in Villameulas, in 1856, by D. José Joaquim Culebras y Morales. In vernal intermittents, the author has seen good effects from the use of three or four slices of lemon sprinkled with sugar. Sometimes yielding to the wishes of the patients, who were afraid of the expense of drugs, or disliked the taste of them, the author prescribed infusion of coffee laced with a little brandy. Intermittents in the hot season required prompter and more efficacious treatment. Bloodletting, both general and local, was called for in many cases, both to allay gastric irritation and to mitigate congestion or hyperæmia of different viscera. In autumnal intermittents, it was often necessary to have prompt recourse to febrifuges, and especially to the sulphate of quinine. It was prescribed at the coming on of the first fit, in order to cut short the disease, and to prevent the pernicious nature of subsequent paroxysms. The quinine was given to the extent of 10 to 12 grains, in pills or solution, and divided into three doses, in the day; sometimes the quantity was increased to a scruple. The writer complains of the vulgar error in which some of those who were cured by the quinine indulged; viz., in their attributing to the medicine, aches and swellings, which were, in reality, due to the fever itself. In cases marked by much irritability, or in which inflammatory symptoms were present, opium or extract of belladonna and tartaric acid were associated with the quinine. The cost of this article, and the liability to the abuse of arsenical preparations, induced the author to make trial of several succedanea to quinine, which we need not enumerate here; and which were little satisfactory in his hands.

The paper is terminated by some questions, and partial replies to them, touching the greater frequency and violence of intermittent fevers during late years than formerly, in that quarter. The author thinks that an explanation may be found in the greater quantity of rain that fell, and the excessive heats; the first causing an overflow of extensive grounds, and the second an evaporation and partial desiccation with a large evolution of miasmata, the effects of which, on the people of that region, might have been increased by the greater susceptibility induced by the general alarm and dread of the cholera, that had prevailed epidemically. This is certainly an addition to the etiology of intermittent fever, as far as relates to predisposing causes.

The division which follows that just noticed, is "*Forensic Medicine.*" It is occupied by a record of the case of a man, who, in a night affray, received an incised and penetrating wound, half an inch in length and two lines in width, in the left groin, on a level with the left branch of the pubis. The details are reported with great minuteness, by D. Jose Cano y Barat, who was assisted throughout by a medico-chirurgical gentleman, acting by authority in that district. Uncertain of the result, and foreseeing that the case might come before a law tribunal, if the wound proved mortal, they asked of the court to be allowed the additional counsel of two other practitioners. The questions for the consultation to determine were, first, of diagnosis, and second, of treatment. There was hemorrhage, which, by a jet per saltum corresponding with the beat at the wrist, when the wound was a little opened so as to allow of the unobstructed discharge of blood, they were assured came from a wounded artery, and most probably the epigastric. There was also a soft shining tumor, imperfectly pyriform, which they considered to be hernia, caused by the protrusion of a portion of intestine, in consequence of its exposure by the wound, and the opening of the fasciæ, which had hitherto retained it in its proper place. The practical questions were: whether they would take up the artery, and reduce the hernia, or leave the wound to heal, by a natural process, while they would endeavor to keep down consecutive inflammation. As regards the first question, the diminished flow of blood after a short time, so that the life of the patient was in no danger from hemorrhage, induced them to forego any attempt at applying a ligature, and incur the risk of unavoidable irritation, if not of enlarging the wound at the same time. It was also determined to let the hernia remain untouched, without any efforts at reduction; and to limit the treatment of the wound to the employment of simple means. Rising inflammation of the affected tissues was to be opposed by the application of two dozen of leeches around the wound, which was dressed with simple cerate, and over this anodyne emollient cataplasms. Entire abstinence from food was also enjoined. The lymphatico-nervous temperament of the patient forbade the use of the lancet. Redness, swelling, and pain prevailed in the groin, along the course of the spermatic cord, and in the corresponding iliac fossa; and the inflammation extended also to the hypogastric and umbilical regions of the left side, even to the hypochondrium, accompanied with traumatic fever, headache, great prostration, &c. This collection of symptoms was thought to prognosticate suppuration, and consequently the presence of diffused phlegmon. The indications were to detract more blood by free leeching, which was practised four times, while emollients were continued to the wound, and the bowels relieved by enemata, with the farther good effect of diminishing the distention of the abdomen. Such was the treatment pursued for four days; on the eighth, pus began to flow freely from the wound; and this was favored by methodical pressure on the parts contiguous. By the sixteenth day, the purulent discharge had ceased, and the hernia also disappeared spontaneously. Cerate to the wound and methodical compression by layers of lint, graduated compresses, and rollers round the body, together with a suspensory bandage to support the scrotum, were now put into requisition; and a nutritive diet was allowed to the patient. The closing of the wound was accompanied by the formation of some proud flesh, which was removed by touching it with the nitrate of silver.

Contrary to our purpose when we first began a notice of this case, we have been led to repeat most of the details given by the narrator, with a view, as we advanced, of presenting to our readers a specimen of Spanish cautious and conservative surgery, and yet not inactive therapeutics. The entire treatment, and the reasons assigned for it, are creditable to the parties in Spain; and convey to us at home a good lesson, and one worthy of consideration in similar emergencies.

The bearing of this case on Forensic Medicine is not at first very clear; but D. Cano y Barat establishes the connection by a series of queries, which follow those on its surgical bearings. He asks: Ought we to give a certificate of health, notwithstanding the state of feebleness which prevented the wounded man from going to work? Ought we to wait until he had entirely recovered his strength, without regard to the greater or less punishment for a day or for an hour, which might be inflicted on his assailant? Finally, as the Health Law is not put in execution, as the judicial body is not endowed with forensic faculties, from whom are we to claim our honorarium, although by the same law we are obliged to perform the duty in defect of such faculties? From what funds are we to be paid? How shall we be sure of our bread?

We lose much of the point of these questions, from our not knowing what is the Spanish penal law; but we glean enough to learn that the medical mind in the Peninsula is fully alive to the just requirements of our profession, in its relations to the officers and courts of law, when its members are charged either directly or impliedly with the performance of official duties *pro h c vice.*

The next department in *La Espana Medica* is a "*Universal or General Review of the Medical Press.*" The first part consists of a synopsis (not merely extracts) of articles in the Spanish medical journals, with the titles of some of which we are thus made acquainted. The first is from "The Medical Age" (*El Siglo Medico*), and its theme, "Is Phthisis Contagious?" The author of the article, Sr. Mendez Alvaro, advocates the affirmative side of the question. We have not room to follow him in his arguments and authorities, but merely repeat the precautions which he lays down on the occasion. They are, 1. To employ as assistants or nurses to the consumptive, those persons who are not members of his family, who are not predisposed to the disease, nor of the age in which its ravages are the greatest. 2. To lodge the consumptive in spacious and well-ventilated houses. 3. Not to allow any person to sleep in the same bed with him. 4. To arrange it so that there shall be alternate sets of nurses, and that no one of them shall be long exposed to emanations from the consumptive patient. 5. No other person to have his wearing apparel, nor to make use of his chamber utensils or close stool. 6. To whitewash the rooms after death, and to have recourse to the well-known means of purification.

Without adopting the opinion of the contagiousness of phthisis pulmonalis, the superintendents of hospitals for the consumptive, to which, by the way, there are strong objections, may take some useful hints from the above precautionary directions.

The other Spanish medical journals noticed in this department of the Espana are the Medico-Chirurgical Courier, and the Medical Union of Aragon (*El Correo Medico-Quirurgico*, and *La Union de Aragon*). In subsequent numbers we find mention made of *La Cronica de los Hospitales; El Semmanario, Medico Espanal, El Eco de los Cirujanos.*

After the synopsis of home journals, follows a similar synopsis of articles from foreign journals—English and French.

The last two departments are the "Official Section," and "Medical Chronicle," which latter includes an announcement of "Vacancies" of the office of attendant surgeon or medico-chirurgeon in certain places, with a specification of salary and probable income.

On a future occasion we may be tempted to take farther notice of Spanish medical journalism, which displays more vitality and energy than a knowledge of the political, commercial, and manufacturing state of Spain would lead us to suspect. The *Espana Medica* is published in a sheet of eight pages quarto, every five days in the month, making six issues in this period, or seventy-two in the course of a year, being 576 pages, equal to 1500 of our octavo pages, and at a subscription price of 40 rials currency, or two dollars per annum.

---

USE OF OTHER MEN'S BRAINS.—Prof. Blackman, the able editor of the Western Lancet, has, in the last number of that periodical, given Dr. Joshua B. Flint, Prof. of Surgery in the University of Louisville, a severe castigation for the very liberal use which he, the said Dr. Joshua B. Flint, made in his introductory lecture, delivered last November, at the Louisville Marine Hospital, of certain sentences and paragraphs, without acknowledging the source from which they were derived. We annex, as a specimen of this wholesale appropriation, the following paragraphs of Prof. Blackman's notice, only adding that Dr. Flint has always prided himself upon being one of the *original* minds of the country, and of belonging to the class of Physicks and Dudleys, and some other illustrious men who never write much, but who, when they do write, write for posterity.

"At p. 21 of the *Introductory*, we find the following, which the reader, not acquainted with Pinel's article in the 5th vol. of the *Dict. des Sciences Medicales* (in 60 vols.), would not for a moment suppose to be but a literal translation:

PINEL.

'Les hôpitaux, á leur origine, servirent plus à satisfaire la bienfaisance pieuse des chrétiens, qu'à perfectionner la médecine, et les études eurent lieu comme aux époques précédentes. L'école d'Alexandrie etait si célébre alors, qu'Amien Marcellin dit que sa fréquentation assidue donnait toutes sortes de droits à l'exercice de la médecine. Une autre ancienne école moins connue que celle d'Alexandrie, et cependant très-florisante, fut celle de Nisapour en Perse, et on doit remarquer que les hôpitaux s'y trouvaient rapprochés des écoles de médecine, avant le temps des Arabes, auxquels on attribue ordinairement l'honneur de cette heureuse idée. Fondée par l'empereur Aurélien, cette école fut composée de médecins grecs qui firent revivre la médecine d'Hippocrate dans tout l'Orient; elle subsista plusieurs siècles; et ce fut là, sans doute, que se formérent Rhazès, Alli-Abbas, Avicenne et les Arabes les plus celèbres. Cette école, qui était chrétienne, était voisne d'un hôpital.'—*Dict. vol. 5th, p.* 365..

FLINT.

'It is certain that hospitals, at the time of their origin, served rather to satisfy the benevolence of Christians than to contribute to the improvement of medicine. The studies employed for this purpose were carried on much as in preceding periods. The school of Alexandria was then so celebrated, that Marcellin assures us, that to have studied there, was enough to secure all the rights and advantages pertaining to the practice of medicine, although it does not appear that anything like clinical studies formed any part of the education of its alumni. There was also at Nisapur, in Persia, another school of medicine, less famous than that at Alexandria, but nevertheless a very flourishing institution. There was established a hospital, attached to a school of medicine, before the time of the Arabian masters, to whom has generally been attributed the honor of that happy idea.

'Founded in 272 by Aurelian, that school was composed of Greek physicians, whose efforts restored the medicine of Hippocrates throughout the East. It continued through many ages, and was, without doubt, the place where were educated Rhazes, Avicenna, and the most celebrated Arabians who were reared in the eastern part of Asia. That school, which was Christian, had near it a hospital.'"—*Introductory*, *p.* 21.

---

SUICIDAL MONOMANIA.—Psychology, connected with and deducible from physiology, is beginning to be better understood, and to take its proper place in general literature, and especially in biography and history. We may adduce among other instances the following observations by Mr. Masson, Professor of English Literature in University College, London, in his "Life of Chatterton." The author had just before been speaking of young Chatterton's conscious sense of great injustice in passing off his own effusions as those of the Monk Rowley of the fifteenth century, whose manuscripts, forged by Chatterton himself, the latter professed to have discovered.

"After all, however, the most material fact in the case remains to be told. Physical causes were at work. Bereft of the amount of actual food, and of other comforts, necessary even with his abstemious habits, to keep body and soul healthily together; wandering about London in a perpetual state of fever and excitement; returning home to write night after night—without rest or sleep—little wonder if he had overstrained his physical capabilities, and if brain and nerve began to fail in their office. Whatever taint of hereditary insanity was in him, derived from the old line of sextons who had jingled in past generations, the keys of St. Mary's Church in Bristol, and walked at midnight through its aisles, and dug the graves of its parishioners, or derived more immediately, from that drunken wild-eyed father, whom he had never seen, but who used to tell his tavern-companions that he believed in Cornelius Agrippa, the necromancer; it had now, at last, come out in a way not to be mistaken. From his childhood there had been symptoms of it; his fits of weeping; his sudden paroxysms of passion; his long reveries, when he gazed at people, without seeming to see them; his frequent mutterings aloud. Not till now, however, had these traits passed the limits of what could be considered compatible with sanity. But now, almost certainly, these limits were passed. Noticing the strange haggard lad, walking about the streets, muttering, perhaps, to himself, or making sudden gestures, or looking at what was passing sometimes vacantly, and sometimes with glances unusually keen and bright; even strangers could not but follow him with their eyes, and wonder who he was, and where he came from. Had the stranger been one accustomed to the ways of the insane, he would, probably, at once have pronounced that his brain was affected. And had the stranger been able, with this idea in his mind, to pursue his inquiries farther, so as to ascertain what peculiar form or species of insanity had taken possession of him, he would have found that it was that which physicians recognize as the suicidal tendency. Physicians, as all know, do recognize this as a form of madness; and though they allow, that a perfectly sane man may commit suicide after deliberately reasoning on the point, they attribute a large proportion of the suicides to the action of a certain specific impulse which reason cannot overcome. In Chatterton's case, as we have seen, there had been premonitory appearances of the existence of this tendency. The idea of suicide had from the first been familiar to him."

Our commiseration for the untimely end of this unfortunate youth must not, however, blind us to the false pride and waywardness of his nature, which, more than actual necessity, subjected him to the gnawings of hunger, and combined with the latter to derange a morbidly sensitive brain, and drive its possessor to self-destruction. The act of the suicide may be the result of a fit of temporary insanity, but no palliation of this nature ought to be adduced for the often habitually wicked disregard of moral and natural laws, and the voluntary nursing a belief in the venial nature of the crime of self-murder, if not in the actual right that a man has thus to dispose of himself. The judgment warped by such wretched sophistry, any slight annoyance, whether of bodily pain, mental excitation, or disappointment, will serve as an excuse for the commission of suicide. Chatterton wanted "the resources to be found in rectitude and gentleness of a mere worldly character." Religion he had none. Professor Masson, in reference to the state of destitution to which the young poet was reduced in London, where he had gone with the hopes of bettering his fortune, makes the following judicious remarks. They contain a lesson good for all times:

"Quiet, plain schólars have lived, before now, in German or in Scotch university towns, on boiled peas-cods for months, on a single guinea a quarter, earned by teaching, without saying much about it. Had youths of this type been in Chatterton's place in London, in August, 1770, they would most probably have survived the crisis. They would have availed themselves gratefully, and yet honestly, of such small immediate aid as those aunts and other relatives, that we hear of so slightly in Chatterton's letters (one of them, a carpenter, who had married one of his aunts) might, perhaps, though poor, have willingly offered at the sharpest moment of the emergency; and even failing that, they would have conquered by sheer patience. How was it then in Chatterton's case, 'the comforts of Christianity' being placed out of the question?"

---

VOICES DIFFERING WITH LOCALITIES.—Experience, says Fetis, has demonstrated that voices, like vineyards, are, in general, distributed in France, by districts. Picardy furnishes finer basses and in greater number than any other province; and almost all the fine basses, which have shone at the opera, and in the other musical establishments, were from that province. Tenors, and particularly those which are called *high-counters*, or counter-tenors, are to be met with in greater number in Languedoc, and especially in Toulouse and its environs, than in any other part of France. The voices of this kind in that country are of singular beauty, and the chance of preserving them after the change, is much more favorable than elsewhere. Lastly, in Burgundy and Franche-Comté, the female voices have more extent and a purer quality than in all the other provinces.

THE DARK SIDE OF WAR.—Colonel Tulloch, one of the two members of the Crimean Board of Inquiry (the other is Sir John McNeill), in a pamphlet recently published, states that the loss from sickness alone, during the winter of 1854–5, in the Crimean army (including what took place at Scutari and during the passage), amounted to thirty-nine per cent. in the infantry, and in eight corps actually amounted to seventy-three per cent.; this being exclusive of men killed in action, or who died of their wounds. By way of contrast it may be mentioned, that, in the naval brigade, which took a prominent part throughout the whole siege, the deaths from sickness were under four per cent. This terrible mortality was four times greater than that which occurred during the Walcheren campaign, whose horrors aroused the indignation of the country, and produced a perfect storm in the Senate. Colonel Tulloch, in eloquent language, points out the causes of this heartless loss of lives: "It was," he says, "no foeman's hand, no blast of pestilence, but from the slow though sure operation of diseases, produced by means, most of which appeared capable, at least, of mitigation."

ARTIFICIAL VICHY WATER.—One of the most celebrated and frequented mineral springs in France, is that of Vichy, the waters of which, with one exception, are thermal; their range of temperature being from 80° F. to 114° F. The visitor to the springs uses the water both as a bath and for drink. There is a large consumption of it in Paris and other parts of the empire, in which case it of course has the temperature of common water. Not only the natural, but also the artificial mineral water of Vichy is largely drunk at a distance from the springs.

The Vichy water, varying somewhat in strength at the different springs, is remarkable for the large proportion of bicarbonate of soda which it holds in solution. It contains, also, but in small quantity, the carbonates of lime and magnesia, sulphate of soda, peroxide of iron and silex, and the organic product, baregine. The curative powers of the Vichy water are highly extolled in gout and in a great variety of chronic diseases, including those of the liver and spleen, and a deranged and weakened state of the digestive organs; also in scrofula, leucorrhœa, irregular menstruation, and engorgements of the uterus, without acute inflammation, ulceration, and cancerous degeneration of this organ. It exerts remarkably beneficial effects in the lithic acid diathesis, and in calculous formations of the lithic acid variety, including red-sand deposits in the urine.

The artificial Vichy mineral water, prepared after the formula of Souberain, may be used under all the circumstances in which the natural mineral water has been found serviceable. We can speak from personal experience of its efficacy in renal disorder of the lithic acid variety, as well as in cases of chronic dyspepsia, in which cardialgia is a predominant symptom. It has no uniformly aperient operation; but sometimes we have found it to act on the bowels, and more particularly when taken warm on an empty stomach.

We are glad to learn that Mr. Frederick Brown, the well-known pharmaceutist, has begun to manufacture the artificial Vichy water; and we do not doubt that, ere long, there will be a great demand for it. Charged as it is so highly with carbonic acid, this water makes an agreeable beverage; or at least to our taste, is barely distinguishable from the ordinary mineral (carbonated) water of the shops.

But while the Vichy mineral water, both natural and artificial, is adapted to the cure or relief of a large circle of diseases, we must not omit to caution against its use in febrile and inflammatory states of the system, and in irritable temperaments; also in cases of plethora with determination to the head, organic affections of the heart, phthisis, and hæmoptysis.

The quantity to be drunk varies from two to four tumblerfuls, or from one to two pints, taken in succession, before breakfast; or divided so that one-half is used early in the morning, and the other at noon, or an hour or two before dinner. In cases of labored digestion with heartburn, half a tumblerful taken an hour or so after a meal gives great relief. When fears are entertained that the stomach, especially in its empty state, will not bear a cold drink, the water may be warmed by immersing the bottle containing it in warm or hot water, so as to make it resemble more nearly that of the springs itself. The first effect of the Vichy water is exciting, and, for the first few days of using it in full quantity, it sometimes gives rise to a sense of fulness with headache and languor; a common effect of most mineral waters of any activity, in the early stage of their administration.

---

After the celebrated *coup d'etat*, which established the position of the present ruler of France, not a word was allowed to transpire in the French Medical Journals respecting the fate or treatment of the wounded. Not a case was related, not a clinical observation made; and as far as could be learned from these journals, such an event, even as regarded its scientific teachings, never occurred; although respecting former outbreaks, no such silence had ever been observed. Matters do not seem to be much better now, the Medical Journals being subjected to the same interdict as the political ones; for instance, of not discussing the mental state of Verger, the assassin of the Archbishop. The editor of the Moniteur des Hôpitaux, after having in a previous number stated his intention of discussing at full the reasons which, in his mind, proved Verger to be insane, has been obliged in a subsequent one to announce that he is under the necessity of abandoning this project.—*Times and Gazette.*

---

ESCULAPIUS THE SON OF APOLLO.—It is said that the great Boerhaave was more proud of his success as a flutist than of his scientific glories. Haller, the universal genius, was an accomplished musician, and delighted in taking his part on the violoncello. Orfila, in place of becoming the founder of modern toxicology, had well-nigh turned his magnificent baritone voice to profit on the stage. M. Bataille, one of the most admired of the singers at the *Opera Comique*, is a doctor of medicine; and a young *agrégé*

of great promise, and possessed of a beautiful tenor voice, has deserted the Academies of Science for that of music. Quite lately too a Mr. Hans, a young man of twenty-one years of age, has made a promising *début* in Lablache's character in Norma, at the *Theatre Italien,* and is engaged for the next season in London. He is the son of the celebrated Rokitansky, of Vienna.—*Ibid.*

---

We have received reports of the graduating classes of the following medical schools:

| | |
|---|---|
| Jefferson Medical College, | 212 |
| University of Pennsylvania, | 149 |
| University of Nashville, | 137 |
| University of New York, | 120 |
| Pennsylvania College, | 58 |
| University of Louisville, | 50 |
| St. Louis Med. College, | 44 |
| College of Physicians and Surgeons, New York, | 37 |
| Miami Medical College, | 31 |
| Medical College of Ohio, | 31 |
| Missouri Medical College, | 31 |
| Starling Medical College, | 18 |
| Massachusetts Medical College, | 17 |

---

Dr. J. C. Nott has been appointed visiting physician to the United States Marine Hospital, at Mobile, and Dr. C. Z. Blaney to the one at Chicago.

---

At the first meeting of the Mississippi State Medical Society, held recently at Jackson, Dr. W. Y. Gadbury, one of our much valued collaborators, was honored with the presidency.

---

In consequence of other engagements, Dr. Goadby has dissolved his connection with the Medical Independent, a Monthly Review of Medicine and Surgery, and Journal of Microscopical Science, published at Detroit, Mich.

WHAT CAN THE MATTER BE?—We observe with great pleasure that not one of the republications of English medical books that have been laid on our table during the past few months, has been *honored* with an American editor—or, at least, the name of such does not appear. The titles of these works may be found on our last page.

---

Dr. Samuel Long, of Springfield, Ill., reports, in the March number of the Northwestern Medical Journal, a case of ovariotomy, from which the patient entirely recovered in three weeks, without a single untoward symptom.

---

The work on Caloric, by the late Dr. Samuel Metcalfe, of this city, formerly of Kentucky, published a number of years ago, by Pickering, of London, where it created quite a sensation at the time, is in course of republication, by J. B. Lippincott & Co., of this city.

---

WELL-DESERVED COMPLIMENT.—At the close of the commencement exercises of the St. Louis Medical College, the graduating class presented to the Faculty of the institution a portrait of Prof. Chas. A. Pope. Dr. Chas. W. Parsons, of Kentucky, represented the class in some pertinent remarks, and was responded to by Prof. J. B. Johnson, on the part of the Faculty.

---

We would call the attention of the profession to the Quarantine Convention, that is to meet in this city on the 13th of May.

---

The late vicar of Sheffield, the Rev. Dr. Sutton, once said to the late Mr. Peech, a Veterinary Surgeon: "Mr. Peech, how is it that you have not called upon me for your account?" "Oh," said Mr. Peech, "I never ask a *gentleman* for money." "Indeed," said the vicar, "then how do you get on if he don't pay?" "Why," replied Mr. Peech, "after a certain time I conclude that he is *not* a gentleman, and then I ask him!"—*Times & Gazette.*

